# Axial pullout strength comparison of different screw designs: fenestrated screw, dual outer diameter screw and standard pedicle screw

**DOI:** 10.1186/s13013-015-0039-6

**Published:** 2015-05-01

**Authors:** Evangelos Christodoulou, Suresh Chinthakunta, Divya Reddy, Saif Khalil, Thomas Apostolou, Philipp Drees, Konstantinos Kafchitsas

**Affiliations:** Asklepios Klinik Lindenlohe, Schwandorf, Germany; Globus Medical Inc., Ave 2560 General Armistead Ave, Audubon, PA 19403 USA; G. Papanikalaou Hospital Exohi, 570 10 Thessaloniki, Greece; Universitätsmedizin Mainz, Langenbeckstr, 1 55131 Germany

**Keywords:** Pullout strength, Fenestrated screws, Biomechanical study, Pedicle screws, Large diameter screws

## Abstract

**Background:**

The pullout strength of pedicle screws is influenced by many factors, including diameter of the screws, implant design, and augmentation with bone cement such as PMMA. In the present study, the pullout strength of an innovative fenestrated screw augmented with PMMA was investigated and was compared to unaugmented fenestrated, standard and dual outer diameter screw.

**Methods:**

Twenty four thoracolumbar vertebrae (T10-L5, age 60 to 70 years) from three cadavers were implanted with the four different pedicle screws. Twelve screws of each type were instrumented into either left or right pedicle with standard screw paired with unaugmented and dual outer diameter screw paired with augmented fenestrated screw in any given vertebra. Axial pullout testing was conducted at a rate of 5 mm/min. Force to failure (Newtons) for each pedicle screw was recorded.

**Results:**

The augmented fenestrated screws had the highest pullout strength, which represented an average increase of 149%, 141%, and 78% in comparison to unaugmented, standard, and dual outer diameter screws, respectively. Pullout strength of unaugmented screws was comparable to that of standard screws, however it was significantly lower than dual outer diameter screws.

**Conclusions:**

Fenestrated screws augmented with PMMA improve the fixation strength and result in significantly higher pullout strength compared to dual outer diameter, standard and unaugmented fenestrated screws. Screws with dual outer diameter provided enhanced bone-screw purchase and may be considered as an alternative technique to increase the bone-screw interface in cases where augmentation using bone cement is not feasible. Unaugmented screws can be left in the pedicle even without cement and provide similar pullout strength to standard screws.

## Background

Pedicle screw fixation is one of the most commonly used forms of stabilization in the thoracic and lumbar spine for trauma, correction of deformity or instability, and fixation in oncologic and fusion procedures [1,2]. However, obtaining adequate purchase with standard pedicle screw fixation remains a challenge in spines with poor bone quality due to complications such as screw loosening, and migration or back-out [3-5]. Several techniques have been proposed to improve the bone-screw interface strength including using larger diameter screws, a cortical bone trajectory (more medial-to-lateral path) [6] and augmentation using bone cement [4,5]. Furthermore, the interface strength may be increased by use of expandable pedicle screws, resorbable polymers, rib grafts, milled bone, and matchstick bone [7].

Fixation of the screw into the vertebral body is traditionally evaluated by determining the axial pullout strength of implanted screws. In the present study, the two most frequently used methods, augmentation with PMMA and large diameter pedicle screws, were tested. Previous biomechanical studies have demonstrated that pedicle screw augmentation using PMMA markedly increases the strength of the bone-screw interface [2-4,8-10].

**Figure 1:**
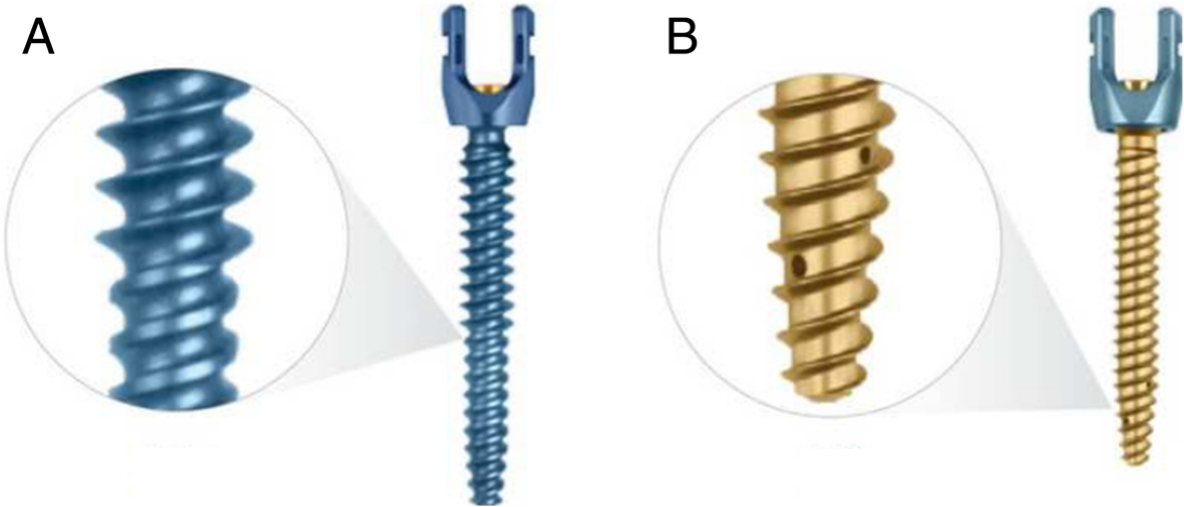
Screw designs: **A)** REVERE® dual outer diameter screw (DOD) with large outer diameter (7.0 mm) and nominal diameter (5.5 mm) at the distal end; and **B)** REVLOKTM fenestrated screw (FS), fully cannulated with four radial screw fenestrations for even 360° cement distribution.

However, few biomechanical studies have compared the pullout strengths of these two frequently used methods for improving the bone-screw interface strength [9,10]. For this study, an innovative fenestrated screw fully cannulated with four radial screw fenestrations and augmented with PMMA (FSP) was compared to a dual outer diameter (DOD) screw, with a larger outer diameter at the proximal end, to optimize the purchase in the cancellous bone (Figure 1). There are also concerns growing regarding the bone-screw interface strength of fenestrated screw (FS) itself compared to a standard screw (SS). This especially arises in situations where the fenestrated screws are left inside the patient once the surgeon determines to have obtained enough purchase and decides against using any additional augmentation. To study the effect of this situation, unaugmented fenestrated screws were compared to standard pedicle screws with regards to pullout strength. Previous studies investigating pullout strength have either used different screw designs [1,3-5,11] compared different bone cements and parameters for augmentation [2,5,12] or have compared different insertion techniques [9-11,13] and have not compared fenestrated screws to dual outer diameter screws in a cadaveric model.

## Methods

### Specimen preparation

Twenty-four thoracolumbar vertebral bodies (T10-L5) from three cadavers (one male, two female and age 60 to 70 years) free of metastatic disease or primary bone disease were used in the study. All cadavers were donators at the Institute of Anatomy at LSU in New Orleans (USA) and were provided for the purpose of this study according to the regulations of the ethics committee. Lateral and anterior radiographs were taken for each specimen to rule out any pathological fractures or other bone lesions. The vertebrae were freed from all muscular attachments. All vertebrae were tested for osteoporosis via dual energy X-ray absorptiometry (DXA). The limit for osteoporosis was set at <0,8 g/cm2 [14].

### Screw placement and cement augmentation

Twelve screws of each type were inserted in the 48 pedicles of 24 vertebral bodies. A pedicle awl was used to perforate the cortex, and a pedicle probe to open the pedicle pathway. A ball-tip probe was used to ensure the pedicle was intact. A 4.5 mm tap was used prior to screw insertion. Pedicle screws were inserted straight into the pedicle, with as minimum convergence as possible [9,13]. In each vertebral body, the SS screw was always paired with a FS screw and the DOD screw was paired with a FSP screw with PMMA on the contralateral side. Screws of same size and length (5.5 mm diameter × 55 mm length) were inserted at each level. However, it must be noted that the nominal diameter (5.5 mm) of the dual outer diameter screws is 1.5 mm smaller than the larger diameter at the proximal end (7.0 mm). The screw heads were removed for each screw to make room for the adapter used for screw pullout. All screws were inserted by hand using a ratcheted screwdriver until approximately 45 mm of the screw length was inside the vertebra, leaving behind 10 mm to accommodate placement of the adapter used for gripping the pedicle screw. Radiographs were used to assess the screw position.

**Figure 2:**
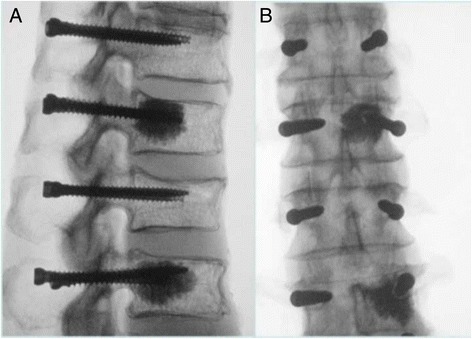
Lateral **(A)** and posterior radiographs **(B)** of one of the specimens showing the screw placement and the injected cement spread through the bone. Placement of screws in straight fashion prevented the screws from coming in contact with each other, which could otherwise compromise the pullout strength.

In the case of fenestrated screws augmented with PMMA, 2 to 3 ml of bone cement [Tecres S.P.A., Verona, Italy] was injected according to the recommended technique [15-17]. Cement mixing was performed according to the labeled instructions and drawn into polypropylene syringes as soon as uniform consistency was achieved. The syringes were then fitted to a luer lock dispensing tip connected to the fenestrated screw to facilitate controlled delivery of bone cement. The location of the radio-opaque bone cement was verified using fluoroscopy. No cement extravasation occurred in any of the vertebra. Since the screws were placed straight into the pedicles, none of the screws on the contra-lateral side came in contact with the cemented fenestrated screws, which could otherwise compromise the pullout strength (Figure 2). The instrumented specimens were tested after 24 hours so that the cement could reach its maximal compressive strength [12].

### Mechanical testing

**Figure 3:**
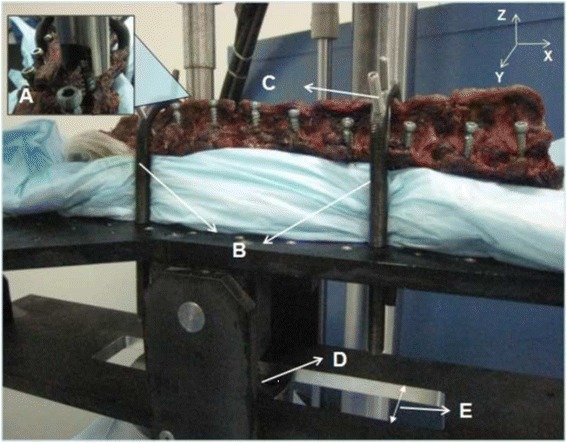
Test fixture for screw pullout test. **A)** Adapter (gripping the pedicle screw head) used for pulling out the screw; **B)** U-clamps hold the specimen in place while allowing rotation of the specimen to align the pedicle to be placed directly in line with the adapter for true axial pullout;**C)** Additional fixation was applied to the lamina (after pedicle screw were inserted and oriented perpendicular to the load cell) to prevent rotation of the specimen during pullout testing; **D)** Load cell of the MTS machine to which the fixture was attached; **E)** Slot that allows for translation in the X-Y direction to perform pullout testing on the left and right side.

After 24 hours, specimens were mounted on a custom-designed fixture attached to the actuator of the test machine (MTS Corporation, Minneapolis, MN). The fixture allowed for rotation in the YZ-plane and adjustment of the XY-plane to ensure vertical pullout alignment (Figure 3). Axial pullout testing was performed at a rate of 5 mm/min was applied. The force to failure was quantified by the test machine and recorded. Load–displacement curves were also recorded.

Failure was defined as the point at which the load peaked and then decreased sharply with increasing displacement. The mean pullout strengths for various screws were calculated. The vertebrae were disarticulated and the pedicles were examined after the completion of the mechanical testing in an attempt to determine the site of failure and any gross physical differences among the four screws were noted.

### Data analysis

In two specimens, the T10 pedicle size was too small resulting in breakage of pedicles. In two other specimens, the L5 vertebra was damaged during pullout testing. These four vertebrae were therefore removed from the study. Therefore, ten screws of each type were used for data analysis. The configuration of each type of screw in each specimen is shown in Table 1. Statistical analysis was performed on raw data using Student *t* test to determine whether axial pullout strengths differed among different screws. Statistical evaluation included paired *t* test between FSP screw versus DOD screws and unaugmented FS screws versus SS screws. For all the other comparisons, unpaired *t* test was used. Significance was set at p < 0.05.

**Table 1:** **Screw pullout strength (numbers in Newton) for every screw type in each vertebral body in the three spine specimens tested**

**Vertebral level**	**Spine specimen 1 64y, male BMD = 0,774 g/cm2**		**Spine Specimen 2 60y, female BMD = 0,649 g/cm2**		**Spine Specimen 3 70y, male BMD = 0,762 g/cm2**	
	**Left pedicle**	**Right pedicle**	**Left pedicle**	**Right pedicle**	**Left pedicle**	**Right pedicle**
T10	1710 (FSP)	1073 (DOD)	R	R	R	R
T11	947 (FS)	868 (SS)	326 (SS)	521 (FS)	582 (SS)	873 (FS)
T12	1628 (FSP)	785 (DOD)	927 (DOD)	1098 (FSP)	989 (SS)	762 (FS)
L1	727 (FS)	858 (SS)	763 (SS)	345 (FS)	1509 (FSP)	620 (DOD)
L2	1588 (FSP)	1067 (DOD)	623 (DOD)	1225 (FSP)	560 (FS)	435 (SS)
L3	605 (FS)	704 (SS)	274 (SS)	302 (FS)	898 (DOD)	1555 (FSP)
L4	1199 (FSP)	800 (DOD)	430 (DOD)	1499 (FSP)	1245 (DOD)	1692 (FSP)
L5	R	R	301 (SS)	265 (FS)	R	R

FS = Fenestrated Screw; SS = Standard Screw; DOD = Dual Outer Diameter Screw; FSP = Fenestrated Screw with PMMA; R = Removed.

## Results and discussion

The unaugmented fenestrated, standard and dual outer diameter screws failed at the bone-screw interface due to bone fracture. For the fenestrated screws augmented with PMMA, entire pedicle was pulled off of the posterior aspect in a few of the specimens, while others failed at the cement-bone interface, where a void was created inside the vertebral body after the removal of the screw/cement section.

**Figure 4:**
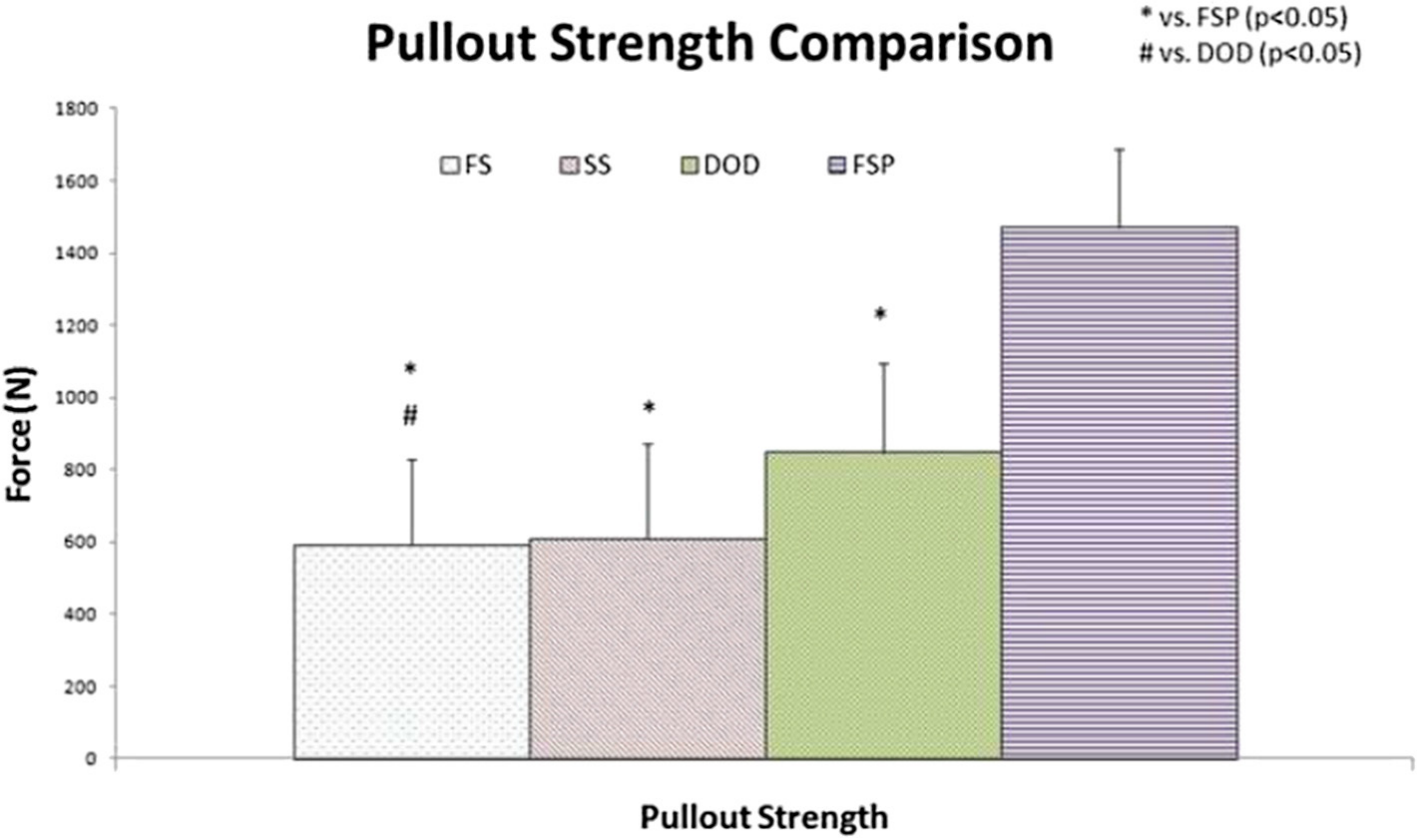
Mean pullout strength. FS = Fenestrated Screw, SS = Standard Screw, DOD = Dual Outer diameter Screw, FSP = Fenestrated Screw Augmented with PMMA.

Overall the mean pullout strengths for each screw type for the 3 specimens tested were: standard screw 610 N (±264), unaugmented fenestrated screw 591 N (±238), dual outer diameter screw 827 N (±274), and fenestrated screws with PMMA 1470 N (±218). These values showed that augmentation with PMMA significantly improved the pullout strength to 149%, 141%, and 78% in compare to the unaugmented fenestrated screw (p = 0,00000000835), standard screw (p = 0,0000000394) and dual outer diameter screw (p = 0,0000328), respectively. No statistical significance was seen between pullout strengths of the standard and fenestrated screws (p = 0,802634562626576). The dual outer diameter screw significantly improved pullout strength compared to the unaugmented fenestrated screw (p = 0,0297). No statistical significance was seen between pullout strengths of the standard and dual outer diameter screw (p = 0,0527). The means and standard deviations for mean pullout strengths for different screws are presented in Figure 4. All the vertebrae had bone mineral density <0,8 g/cm^2^ that indicated osteoporosis [14].

Use of standard pedicle screws in the osteoporotic spine remains a challenge due to the mechanical instability of the bone-screw interface. Numerous techniques have been proposed to increase the bone-screw interface strength including bicortical purchase, pedicle undertapping and offset laminar hooks [7]. Furthermore, the interface strength may be increased by use of expandable pedicle screws, resorbable polymers, rib grafts, milled bone, and matchstick bone [7]. In the present study, the pullout strength of the fenestrated screw augmented with PMMA was compared to the dual outer diameter screw, which has a larger diameter at proximal end and a nominal diameter at the distal end. Furthermore, the unaugmented fenestrated screw was compared to a standard pedicle.

In the present study, pedicle screws were inserted at a straight angle as opposed to the standard angled screw insertion. Sterba et al. (2007) have demonstrated that straight screw insertion results in a more stable pedicle screw construct as opposed to angled screw insertion technique [18]. The authors noted that angled screw insertion results in more data scatter. In addition, this insertion technique holds the straight screw close to the cortical region of the vertebra at three regions: 1) at the insertion point, 2) across the pedicle, and 3) at the end point [18]. This justifies the implantation of pedicle screws in the present study across the axis with as minimum convergence as possible rather than along the axis. From a clinical viewpoint, insertion of the pedicle screws in a straight fashion is certainly more practical as it does not require wide dissection, retraction, or excision of paraspinal musculature to achieve screw insertion along transverse pedicle angles that can range up to 38° from midline [18]. In addition, this technique also help prevent overlapping of screws which would result in false data.

Augmentation using PMMA resulted in highest pullout strength in the three specimens tested. Over all, pedicle screw augmentation with PMMA increased the pullout strength to 241% compared to that of standard screws. Other biomechanical studies have demonstrated that pedicle screws augmented with PMMA improve initial fixation strength and fatigue strength of instrumentation in the osteoporotic spine [2,3,8,9]. Cook et al. (2004) in a biomechanical study comparing cemented and uncemented expandable screws reported a 250% increase in mean pullout strength with a cemented expandable screw compared to an uncemented screw in the human thoracolumbar spine [3]. Another biomechanical study that evaluated pullout strength of screw augmented with calcium sulfate or PMMA reported an increase of 167% and 199% with calcium sulfate and PMMA, respectively, compared to unaugmented screws [2]. The difference in results could be mainly attributed to straight screw insertion technique used only in this study, apart from test setup and screw designs. Thus the novel fenestrated screws augmented with PMMA may be useful for pedicle screw fixation in patients with poor bone quality.

A commercially available radiopaque PMMA was used to strengthen fenestrated screws. PMMA is typically used to increase bony purchase [2,9]. One disadvantage of screw augmentation with PMMA is neurologic injury resulting from direct compression of neural elements by extravasation or thermal effects of cement curing [4]. However, more recently PMMAs used in spinal surgery are radiopaque and have reduced exothermic polymerization reaction to reduce tissue necrosis and nerve damage in the event of leakage [19].

In each vertebral body, the standard screw was always paired with a fenestrated screw and the dual outer diameter screw was paired with a fenestrated screw augmented with PMMA on the contralateral side, to minimize the potential differences in bone mineral density and be able to compare the pullout strengths of the two frequently used methods of fixations. In addition all the vertebrae used had a bone mineral density less than 0,8 g/cm2, it means in effect they were all osteoporotic [14]. Dual outer diameter screws may be used as a method of supplementing, replacing or augmenting screw purchase after the failure of primary spinal instrumentation and in the osteoporotic spine. In the three specimens tested in this study, the mean pullout strength of dual outer diameter screw was always higher than the standard and unaugmented fenestrated screw. Over all, dualouter diameter screws increased the pullout strength to 140% and 136% compared to unaugmented fenestrated and standard screws, respectively. However, this increase was significant only with respect to unaugmented fenestrated screws. Previous biomechanical studies have shown that larger diameter screws offer significantly increased fixation strength than standard screws [9-11]. Wittenberg et al. (1993) concluded that a 1-mm increase in screw diameter significantly increases axial pullout strength [10]. Polly et al. (1998) showed that for pedicle salvage, increasing screw diameter causes the greatest restoration of strength [11]. Advantage of the DOD screws compared to SS screws with the same diameter as the outer proximal DOD diameter is the preparation of the pedicle canal with the distal self-tapping threaded portion which has a smaller outer diameter and allows a more central impantation of the screw [20]. This does not require separate initial tapping, which would reduce the pull-out strength [21,22]. For these reasons, the DOD-screws reduce the risk of pedicle breakage especially in osteoporotic bone [22-24] and increase the screw/pedicle quotient, which is crucial for the stability of the spinal fusion. Thus increasing the diameter of the pedicle screw may be a viable alternative to improve bone-screw interface strength, especially in situations where augmentation using bone cement is not feasible such as disruption of bony margins due to screw placement.

Furthermore there was no significant difference between the pullout strength of the unaugmented fenestrated screws and the standard screws. Previous studies [21] have shown that tapping or removing the screw reduces the pullout strength. According to the present study an unaugmented fenestrated screw can be installed in the pedicle without cement if necessary, providing pullout strength in osteoporotic bone similar to the standard screw.

Limitations resulted from the experimental setup. Factors as muscle strength, body weight and height were not taken under consideration. Moreover this study did not analyze the age- and sex-specific differences. The use of cadaver specimens also brings restrictions. Despite rapid action and optimal humidification during the experiment, there was a certain degree of autolysis that cannot be prevented. Furthermore the stabilizing effect of the intervertebral discs, ligaments and muscle surrounding the spine in vivo was removed before the experiment. As a result changes in biomechanical properties cannot be excluded. Other limitations arise from the assumption that the measured bone density of vertebral body is equivalent to the bone density of the pedicle [25]. According to current studies, bone density in the vertebral body is up to six times higher than in the pedicle bone in healthy subjects. Due to the small number of specimens (n = 24) and the exclusive use of osteoporotic vertebral bodies (BMD < 0.8 g/cm) no correlation between bone density and pullout strength has been determined. In addition, the vertebrae were not classified by osteoporosis degrees.

## Conclusions

The novel fenestrated screws augmented with polymethylmethacrylate resulted in a significant increase in the axial pullout strength compared to dual outer diameter, standard and unaugmented fenestrated screws. This suggests that the novel fenestrated screws augmented with PMMA may be useful for pedicle fixation in patients with poor bone quality. The dual outer diameter screws improved the pullout strength compared to the standard and fenestrated screw and may be considered as an alternative technique to increase the bone-screw interface, in cases where augmentation using bone cements is not feasible. The novel fenestrated screw without cement augmentation demonstrated pullout strengths comparable to the standard pedicle screw.

## Abbreviations

PMMA: polymethylmethacrylat
T: thoracic
L: lumbal
N: Newton
FSP: augmented fenestrated screws
DOD: dual outer diameter screws
FS: unaugmented fenestrated screws
SS: standard pedicle screw
Mm: milimeter
Ml: mililiter
DXA: dual energy X-ray absorptiometry
mm/min: milimeter/minute
BMD [g/cm2]: Bone mineral density [gram/square centimeter]
Y: years old
